# Use of Rituximab in Management of Rapidly Progressive Glomerulonephritis

**DOI:** 10.7759/cureus.6820

**Published:** 2020-01-30

**Authors:** Ivan Cancarevic, Bilal Haider Malik

**Affiliations:** 1 Internal Medicine, California Institute of Behavioral Neurosciences and Psychology, Fairfield, USA

**Keywords:** rituximab, rapidly progressive glomerulonephritis, anti-gbm, goodpasture syndrome, lupus nephritis, iga nephropathy, vasculitis

## Abstract

Rapidly progressive glomerulonephritis (RPGN) is a form of glomerulonephritis characterized by loss of renal function within weeks. Although a variety of underlying causes can trigger RPGN, the ultimate pathologic mechanism is the podocyte and epithelial activation leading to the crescent formation. Rituximab has been increasingly and successfully used for autoimmune conditions in recent years. Treatment of RPGN is based on the underlying condition, but specific clinical guidelines are lacking. In this article, we have tried to establish the role of rituximab in the management of patients with RPGN. All the studies we have used were found in the PubMed database, limited to studies involving adults. Animal studies and studies involving the pediatric population were excluded. The currently available literature does not support switching to rituximab as the first-line agent. It has failed to prove consistently superior to other medications. However, combined with other commonly prescribed treatment regimens, namely corticosteroids, with or without cytotoxic drugs, rituximab has shown efficacy in many studies. Therefore, we have concluded that the most prudent use of rituximab in patients with RPGN would be in those with disease refractory to standard management with corticosteroids and cytotoxic drugs or in those who have intolerable side effects. We believe that clinicians should keep reporting any cases of RPGN treated with rituximab so that a more clear pattern emerges and more exact treatment guidelines can be made.

## Introduction and background

Rapidly progressive glomerulonephritis (RPGN), also known as crescentic glomerulonephritis, is a form of glomerulonephritis characterized by rapid loss of renal function, usually within weeks [1]. It most commonly occurs secondary to a systemic disease with renal involvement, although idiopathic cases have been described. Three forms are generally defined according to the pathophysiologic mechanism leading to renal injury [1-2]. Immunofluorescence can be used to distinguish them [1-2]. Type 1 refers to the anti-glomerular basement membrane (anti-GBM) disease with linear deposits, the most famous of which is Goodpasture syndrome [2]. Type 2 refers to immune-mediated forms of the disease characterized by granular antibody and complement deposits [2]. Type 3 refers to cases characterized by no or few immunoglobulin deposits (e.g., antineutrophil cytoplasmic antibodies (ANCA)-associated vasculitis)[2]. Ultimately, all three types result in the activation of podocytes and epithelial proliferation in Bowman’s capsule and formation of crescents, which can be visualized on light microscopy [3-5]. Clinically, RPGN can be defined as a syndrome of rapid loss of renal function with evidence of glomerular inflammation (e.g., hematuria), rapidly decreasing glomerular filtration rate (GFR), early oliguria or anuria, with or without hypertension, edema, and proteinuria [6]. The overall prognosis depends on many factors, including the underlying cause, creatinine levels at presentation, percentage of glomerular involvement, and treatment delay [2,4].

Developing detailed treatment guidelines for RPGN is difficult, in part, because of the variable etiology of the disease. Corticosteroids, immunosuppressive treatments, plasma exchange, and anti-B monoclonal antibodies have been tried with varying degrees of success. No treatment has consistently shown excellent response rates in patients diagnosed with RPGN [2,5]. With early treatment, many patients achieve at least partial remission [2,5]. Traditionally, the most commonly recommended treatment strategy for remission induction has involved the use of corticosteroids with or without the addition of immunosuppressants, such as cyclophosphamide [7]. Plasma exchange therapy has been used for patients who failed to respond to corticosteroids and immunosuppressants [7]. An exception to the rule is the anti-GBM disease, where plasma exchange is generally performed early [7]. Following the induction therapy, maintenance therapy with less toxic agents is given [7]. Such protocols have resulted in a 50-70% 5-year survival rate, with relapse rates as high as 50%, depending on the cause [7]. Treatment duration and recurrence markers have not been clearly established [1].

Rituximab was first approved for clinical use in the late 1990s and has since been one of the most commonly used and important medications in clinical practice [8]. It is most commonly associated with the treatment of hematologic malignancies, although nowadays, its use in autoimmune diseases has been increasing [8]. As a monoclonal antibody that targets CD20 on B cells, it has been used at least somewhat successfully for a variety of conditions mediated by B cells, including rheumatoid arthritis, Sjögren's syndrome, and idiopathic membranous nephropathy [9-11]. As is the case with cytotoxic drugs, the use of rituximab reduces the necessary dosage of corticosteroids and decreases the likelihood of side effects associated with corticosteroid use [11-12]. It is generally well tolerated compared to most cytotoxic drugs, although adverse reactions, including serious ones, have been reported (acute respiratory distress syndrome, myocardial infarction, toxic epidermal necrolysis) [11-12]. The evidence to suggest long-term safety is still lacking due to the relative novelty of the drug [11-12].

Rituximab has been used in the treatment of RPGN, although the recommendations for its use have not been clearly laid out [2]. Some of the most detailed treatment guidelines were published by Arimura et al. and did recommend some uses of rituximab, especially for RPGN caused by ANCA-associated vasculitis [13]. In this article, we are going to explore whether the currently available literature supports recommending the use of rituximab for the treatment of RPGN.

## Review

The articles used for this review have been found in the PubMed database. Full quality assessment of individual articles could not be performed due to limited access to some of the articles.

Considering that the causes of RPGN can be divided into three groups, we will be analyzing the treatment response in each group.

 

Anti-GBM Disease

 

Anti-GBM disease usually presents as RPGN with the formation of glomerular crescents. Alveolar hemorrhage occurs in about half of the cases [14-16]. The standard treatment involves plasmapheresis, corticosteroids, and cytotoxic drugs [16]. Despite such treatment, only about a third of patients retain kidney function for six months [15]. Marques et al. reported an overall five-year survival rate of 92% with older age, female sex, and higher serum creatinine at the presentation being identified as indicators of worse outcomes [17]. Several studies have been done to assess whether rituximab could lead to better prognosis in patients with anti-GBM disease. Touzot et al. analyzed the outcomes of eight patients in whom rituximab was added to standard therapy within two months of diagnosis [18]. Seven of them achieved complete remission and, after a mean follow-up of 25.6 months, renal survival was 75% [18]. GFR improvement was not observed in response to rituximab therapy [18]. Uematsu-Uchida et al. reported a single case of a good response to treatment with rituximab, steroids, and plasma exchange [19]. Syeda et al., similarly, found the same treatment combination (rituximab, steroids, plasma exchange) effective in a single case [20]. Kaegi et al. analyzed the safety and efficacy of rituximab in multiple immune-mediated disorders [21]. The study included three patients with anti-GBM disease [21]. After a follow-up of 33-49 months, anti-GBM antibodies became undetectable in all three patients, and only one of the patients remained dialysis-dependent [21]. They reported no adverse effects [21]. Heitz et al. studied five patients with anti-GBM disease who were treated with rituximab as first-line therapy, rather than cyclophosphamide [22]. Although anti-GMB antibodies became undetectable in all patients, and pulmonary improvement was significant, no improvement in renal outcomes was observed [22]. They also reported no major adverse reactions [22]. Schless et al. reported treating two patients with the refractory anti-GBM disease with a single dose of rituximab [23]. Renal recovery was not observed in either of the patients, and one of the patients developed leukoencephalopathy [23]. Sauter et al. reported a case of an anti-GMB disease relapse 18 months after renal transplantation [24]. Rituximab was added after the failure of first-line treatment [24]. Anti-GBM antibody levels rapidly decreased, but there was no effect on renal function, and dialysis had to be initiated [24].

Overall, 13 patients were included in the three studies that showed promising responses to treatment with rituximab, and only three of them became dialysis-dependent. Eight patients were involved in the studies that showed no improvement in renal function. However, in those studies, rituximab was never initiated both early and together with plasmapheresis, steroids, and cytotoxic drugs. Decreased levels of anti-GBM antibodies were typically significantly decreased, regardless of renal recovery. When rituximab was initiated together with standard first-line treatment, multiple studies have shown promising results regarding renal recovery, especially compared to the prognosis reported with standard treatment alone. It should be noted that the total number of patients in all the studies is low, and it is difficult to draw conclusions about the efficacy of rituximab. One clearly observed pattern is that, if used, rituximab should not be used as a stand-alone treatment. Also, due to a lack of specific guidelines for rituximab use in anti-GBM disease, the dosage received by patients in the reported studies varied significantly. In order for a more detailed analysis, a larger number of patients would need to be studied. Due to the rarity of the condition, large clinical trials may not be realistic, and we would, therefore, encourage clinicians to keep reporting any anti-GBM disease cases treated with rituximab so that a larger review can be performed once more data are available.

 

Lupus Nephritis and IgA Nephropathy/Henoch-Schonlein Purpura

The systematic lupus erythematosus (SLE) is one of the most common and well-known causes of immune complex-mediated RPGN. Kidneys are involved in up to 50% of patients with SLE [25]. Corticosteroids and immunosuppressive drugs are the standard first-line agents [25]. The use of rituximab in SLE has been more widely studied than that in anti-GBM disease, and more data are available. It has typically been used in cases of treatment resistance. For this review, we have limited our search to the studies involving at least 50 patients. Cases of severe renal disease in patients with IgA nephropathy/Henoch-Schonlein Purpura (HSP) have also been reported, and due to their similarity in pathogenesis with SLE, we are going to look at those studies as well. In 2012, Rovin et al. published the results of a randomized, double-blind, placebo-controlled phase III trial (LUNAR) [26]. Patients were given either rituximab or placebo in addition to mycophenolate mofetil (MMF) and corticosteroids [26]. Rituximab treatment resulted in improved laboratory findings (complement and antibody levels) [26]. The clinical response rates were higher compared to placebo but failed to reach statistical significance [26]. Neutropenia, leukopenia, and hypotension were more commonly seen in patients treated with rituximab, although serious side effect rates were similar between the groups [26]. Condon et al. proposed a steroid-avoiding protocol and concluded that oral steroids could be safely avoided in favor of rituximab [27]. Ramos-Casals et al. performed a systematic review that analyzed the outcomes of 106 patients treated with rituximab [28]. Complete or partial remission was seen in 69%, and they concluded that those findings would support rituximab use in lupus nephritis (LN) [28]. They noted that non-responders were more likely to be young, black, and lacking CD19(+) depletion [28]. Tsanian et al. looked at general response rates to rituximab among patients with refractory SLE [29]. Importantly, they noted that there was no increased risk of infectious or other adverse effects among patients treated with rituximab [29]. Lafayette et al. analyzed the effects of rituximab on the management of severe IgA nephropathy and renal dysfunction [30]. While rituximab did successfully deplete B cells, it did not lead to improved GFR or proteinuria [30]. Moreover, it did not produce a reduction in antibody levels [30]. However, there have been multiple reports of patients achieving excellent responses with significant clinical improvements, including a study following renal transplantation [31-33]. The largest study involving adult patients with HSP that we have found was done by Maritati et al. and included 22 patients, with a median follow-up time of 24 months [34]. Rituximab administration led to clinically significant decreases in proteinuria, CRP level, and required prednisone dose [34]. GFR remained stable, and the treatment was well tolerated [34]. One patient died after five years [34].

The LUNAR study did not find strong evidence in favor of rituximab use in LN. Patients given rituximab suffered more side effects (although none were considered serious), and the improvement in their response rates failed to reach statistical significance. Meanwhile, some newer systematic reviews are pointing towards a higher efficacy of rituximab, although the response rates seemed to vary across patient populations. Rituximab was not associated with high rates of serious adverse reactions in any of the studies. The majority of the studies we have found suggest high efficacy of rituximab in patients with HSP/IgA nephropathy. Only one study (by Lafayette et al.) seems to contradict that. Serious adverse reactions were not reported in any of those studies. As of today, there does not appear to be enough evidence to recommend using rituximab as a first-line agent in LN in all patients. However, considering the low incidence of serious adverse reactions and the high risk of development of end-stage renal disease (ESRD), rituximab can be considered as a second-line treatment for patients who fail to respond to first-line treatments. When it comes to IgA nephropathy and HSP, it appears like rituximab is a reasonable alternative management option, especially for patients with refractory disease or those who require long-term steroid use. The amount of data is limited, and we would encourage clinicians to report any new cases.

 

Vasculitis with Renal Involvement

 

Vasculitis, especially granulomatosis with polyangiitis, has been associated with RPGN. While immunosuppressive treatments, such as cyclophosphamide and corticosteroids, remain the most frequently used drugs in the management of ANCA-associated vasculitis, rituximab has also been studied, and it is approved for remission induction [35-36]. It also appears to be as effective as maintenance therapy [35-36]. Fewer studies have looked into its use for severe renal dysfunction specifically. Geetha et al. found that for patients with ANCA-associated vasculitis with GFR < 20 ml/min/1.73m(2), the addition of cyclophosphamide did not result in improved clinical outcomes compared to rituximab and glucocorticoids alone [37]. Another study by Geetha et al. found no difference between rituximab and cyclophosphamide for remission induction, both when it comes to response rates and adverse effects [38]. Glucocorticoids were used as an additional treatment in both groups [38]. Murakami et al. also found rituximab to be a good alternative agent [39]. Berden et al. found that tubular atrophy on kidney biopsy was an independent prognostic factor after rituximab therapy [40]. They also found evidence of T cell involvement and, therefore, recommended looking into therapies targeting T cells in addition to rituximab [40]. ANCA-associated vasculitis is also the most common etiology of RPGN in the elderly [41]. They tend to respond to the same treatments as younger patients; however, the risk of side effects is higher, and age is considered an independent risk factor for adverse clinical outcomes [41-42].

These studies clearly point to the non-inferiority of rituximab compared to cyclophosphamide in the treatment of severe glomerulonephritis caused by ANCA-associated vasculitis. Interestingly, the combination of rituximab and cyclophosphamide did not yield improved clinical outcomes. Glucocorticoids were used in all patients in all the studies we have looked at. Tubular atrophy was found to be an important prognostic factor following rituximab therapy. The currently available literature does not support switching from cyclophosphamide to rituximab as the first-line treatment option. However, rituximab is clearly effective and should be considered as a second-line treatment for patients who fail to respond to cyclophosphamide or who experience significant adverse reactions to cyclophosphamide. As recommended by Berden et al., therapies targeting T cells should be explored in the future.

## Conclusions

RPGN denotes a severe renal dysfunction that develops over weeks or even days as a result of some form of autoimmune injury to the glomerulus. Rituximab, a monoclonal antibody against CD20 on B cells, has been used successfully in multiple neoplastic and autoimmune conditions previously and we wanted to find out if it was a reasonable treatment option for patients with RPGN. It has generally been successful in achieving B cell depletion but has not proved to be universally superior to other recommended treatments as far as clinical outcomes are concerned. In anti-GBM disease, multiple articles show promising results, but the evidence is still scarce. Patients with IgA nephropathy/HSP tend to respond well to rituximab, and it seems to be a reasonable alternative treatment agent. The data for lupus nephritis is somewhat conflicting and should generally be reserved for patients with refractory disease. For patients with RPGN due to vasculitis, rituximab appears to be comparable to more commonly prescribed cyclophosphamide. It was well tolerated overall. Taking into consideration the significant costs associated with rituximab use and lack of clear evidence of clinical superiority, we recommend reserving it for use in patients who fail to respond to initial treatment or who have intolerable side effects to it.
